# A novel necroptosis-related lncRNAs signature effectively predicts the prognosis for osteosarcoma and is associated with immunity

**DOI:** 10.3389/fphar.2022.944158

**Published:** 2022-08-29

**Authors:** Binfeng Liu, Chengyao Feng, Zhongyue Liu, Chao Tu, Zhihong Li

**Affiliations:** ^1^ Department of Orthopaedics, The Second Xiangya Hospital, Central South University, Changsha, Hunan, China; ^2^ Hunan Key Laboratory of Tumor Models and Individualized Medicine, The Second Xiangya Hospital of Central South University, Changsha, Hunan, China

**Keywords:** osteosarcoma, necroptosis, lncRNAs, prognosis, immunity

## Abstract

**Background:** Necroptosis is closely related to tumorigenesis and development. Accumulating evidence has revealed that long non-coding RNAs (lncRNAs) are also central players in osteosarcoma (OS). However, the role of necroptosis-related lncRNAs in OS remains unclear. In the present study, we aim to craft a prognostic signature based on necroptosis-related lncRNAs to improve the OS prognosis prediction.

**Methods:** The signature based on necroptosis-related lncRNAs was discovered using univariate Cox, least absolute shrinkage and selection operator (LASSO), and multivariate Cox regression analysis. The prognosis efficiency of the signature was then estimated by employing various bioinformatics methods. Subsequently, immunological analysis and Gene Set Enrichment Analysis (GSEA) were used to explore the association between necroptosis-related lncRNAs with clinical outcomes and immune status. More importantly, several necroptosis-related lncRNAs were validated with RT-qPCR.

**Results:** Consequently, a novel prognosis signature was successfully constructed based on eight necroptosis-related lncRNAs. Meanwhile, the novel necroptosis-related lncRNAs model could distribute OS patients into two risk groups with a stable and accurate predictive ability. Additionally, the GSEA and immune analysis revealed that the necroptosis-related lncRNAs signature affects the development and prognosis of OS by regulating the immune status. The necroptosis-related lncRNA signature was closely correlated with multiple anticancer agent susceptibility. Moreover, the RT-qPCR results indicated several necroptosis-related lncRNAs were significantly differently expressed in osteosarcoma and osteoblast cell lines.

**Conclusion:** In this summary, a novel prognostic signature integrating necroptosis-related lncRNAs was firstly constructed and could accurately predict the prognosis of OS. This study may increase the predicted value and guide the personalized chemotherapy treatment for OS.

## Introduction

Osteosarcoma (OS) is the most prevalent primary bone cancer in children and adolescents, caused by mesenchymal stem cells or osteoclasts (Rodriguez et al., 2012; Tao et al., 2014). OS accounts for about 0.2% of all malignancies with an incidence rate of 3-4 people per million, of which children and adolescents account for about 70% (Qin et al., 2020; Sadykova et al., 2020). Before the 1970’s, surgical resection was the only effective treatment for OS, and the 5-year survival rate was reported to be about 20% (Ohno et al., 1975). In recent years, with the emergence of neoadjuvant chemotherapy, the prognosis of patients with localized OS has been dramatically improved. The 5-years survival rate has increased to 60–70% (Harrison et al., 2018). Currently, the main treatment for OS includes surgery, chemotherapy, radiation therapy, immunotherapy, and targeted therapy (Harrison et al., 2018). Unfortunately, owing to the solid invasive ability, early metastasis, and delayed diagnosis of OS, patients with metastasis or recurrence still have a poor prognosis. Their 5-year survival rate is less than 25% (Kempf-Bielack et al., 2005). It is well known that distal metastasis is a significant cause of death in OS patients (Goričar et al., 2014). Early identification of high-risk patients and early active intervention measures are crucial to improving the overall prognosis of OS However, the exact molecular mechanism of OS pathogenesis is still unclear, and the predictive ability of traditional clinical markers has limited predictive power. Therefore, it is urgent to search for a novel diagnostic and prognostic biomarker for patients with OS.

Necroptosis, a new form of cell death resembling, is characterized by three essential proteins: receptor-interacting protein kinase 1/3 (RIP1 and RIP3) and mixed lineage kinase-like protein (MLKL) (Marshall and Baines, 2014; Galluzzi et al., 2018). As the host defense gatekeeper against pathogen invasion, the dysregulation of necroptosis is a crucial factor in many inflammatory disorders. Previous research has demonstrated that necroptosis plays a vital part in various inflammatory diseases, such as atherosclerosis, acute kidney injury, and inflammatory response syndrome (SIRS) (Grootjans et al., 2017; Tonnus et al., 2019). Recently, growing evidence points to the vital role of necroptosis in tumorigenesis and metastasis, suggesting that targeted necroptosis has the potential as a novel therapeutic strategy (Yan et al., 2022). For instance, shikonin plays an anti-tumor effect in OS by elevating the expression levels of RIP1 and RIP3 and then activating cell necroptosis (Fu et al., 2013). As a form of non-coding RNA, long non-coding RNA (lncRNA) can alter tumor cell necroptosis by controlling gene expression in several ways, including epigenetic, transcription, and post-transcription levels (Quinn and Chang, 2016; Chen Z. et al., 2020). For example, Lnc00176 down-regulates target mRNA, such as miR-9 and miR-185, leading to necroptosis activation of hepatocellular carcinoma cells (Jiang et al., 2021). In addition, the potential of necroptosis-related lncRNAs as biomarkers in tumor prognosis prediction and therapy is currently being evaluated. Zhao et al. (2021) confirmed that necroptosis-related lncRNAs signature could improve the prognosis prediction and help guide individual treatment in Gastric Cancer. However, necroptosis-related lncRNA has not been mentioned in OS studies, and the value of necroptosis-related lncRNAs signature as potential prognostic markers or therapeutic targets for OS is still unclear.

In this study, we aimed to screen out independent prognostic necroptosis-related lncRNAs based on the gene expression data and clinical information of OS in public databases and constructed a novel necroptosis-related lncRNA prognostic signature for OS, providing insights for evaluating the clinical prognosis of OS. Additionally, we tentatively explored the potential molecular mechanism of necroptosis-related lncRNAs included in the prognostic models through Gene Set Enrichment Analysis (GSEA). Finally, immune infiltration and drug sensitivity analysis were utilized to further recognize the connections between necroptosis and tumor immune infiltration status, improving the understanding of tumor microenvironment and OS treatment. Hence, the results of our study would provide new insights for exploration of the molecular mechanism of OS pathogenesis and development, as well as provide a new therapeutic strategy for OS treatment in the future.

## Materials and methods

### Data source

The RNA sequencing (RNA-seq) and the corresponding clinical characteristics of OS come from The Therapeutically Applicable Research to Generate Effective Treatments database (TARGET; https://ocg.cancer.gov/programs/target) (Amaravadi et al., 2019), and the RNA-seq of normal tissues come from The Genotype-Tissue Expression database (GTEx, https:// www.gtexportal.org/home/) (Consortium, 2020). The transcriptome data (FPKM values) as mentioned above were all downloaded from The University Of California Santa Cruz (UCSC) Xena Hub datasets (https://xenabrowser.net/) and normalized by log2 (x+1) (Goldman et al., 2015). In total, 396 cases of normal muscle tissue and 88 cases of OS tissue were included in this study to identify necroptosis-related lncRNA. After removing the samples with missing survival data, 84 OS samples were retained for subsequent analyses. The clinical data of patients with OS was also extracted from the UCSC database. Supplementary Table S1 shows the clinical characteristics of OS patients included in this study.

### Necroptosis-related lncRNAs

The necroptosis-related genes in this study were extracted from previous reports about necroptosis (Zhao et al., 2021). The expression matrix containing 67 necroptosis-related genes and all lncRNAs was extracted for subsequent analysis. Subsequently, Pearson correlation analysis was performed between necroptosis-related genes and lncRNAs in OS. The lncRNAs with correlation coefficient |R^2^| > 0.3 and *p* < 0.001 were considered as necroptosis-related lncRNAs (Li X. et al., 2020; Tang et al., 2021). Finally, the R package “limma” was applied to detect the differentially expressed necroptosis-related lncRNAs between OS tissue and normal tissues with FDR<0.05 and |log2FC|≥1.

### Construction of necroptosis-related lncRNAs model

To explain the prognostic significance of necroptosis-related lncRNAs systematically and comprehensively in OS, we employed 84 patients with overall survival data to build prognostic signatures. For OS patients, we extract survival data, including survival time and survival status, and merge them with the expression files of necroptosis-related lncRNAs. The necroptosis-related lncRNAs associated with overall survival were screened out through univariate COX regression analysis and drew a forest plot (*p* < 0.05). Subsequently, they were incorporated into LASSO and the multivariate COX regression analysis to seek independent prognostic necroptosis-related lncRNAs for constructing a precise prognosis signature. The risk score was calculated as $\mathrm{RiskScore}\sum_{k=0}^{n}coef\;\left(lncRNA\;k\right)\text{* }exp\left(lncRNA\;k\right)$ . “*n*” represents the number of pyroptosis-related lncRNA, coef (lncRNA k) represents the coefficient of each lncRNA in the signature, and exp (lncRNA k) represents the lncRNA expression level. The risk score was obtained by multiplying and accumulating the two groups. Patients with OS were separated into high- and low-risk groups according to their median risk score. The predictive value was estimated using Kaplan-Meier (K-M) survival analysis. The Receiver operating characteristic (ROC) curve was drawn, and the area under curve (AUC) value was calculated to assess the predictive power of the prognostic signature.

### Prognostic and independent analysis

Univariate and multivariate analyses were conducted to explore whether the established necroptosis-related lncRNAs prognostic model was an independent prognostic factor for OS. Subsequently, the OS patients were divided into different clinical subgroups according to the clinical data, including age (≥16, < 16), gender, and metastatic status. To further evaluate the independent predictive ability of the novel model, the K-M survival curve was conducted one by one to compare the prognosis differences of two risk groups in each subgroup.

### Nomogram and calibration

To further clarify the association between the novel signature and the prognosis of OS, we constructed a nomogram and corresponding calibration curves. A nomogram that included different variables, such as age, gender, and metastasis, was established to predict the survival possibility of OS patients after 1-, 2-, and 3-years. The calibration curve was used to validate the nomogram’s prediction performance between different patient groups. The R software package “rms” was utilized to build nomograms and calibration curves.

### GSEA

GSEA can calculate the enrichment score of a specific gene set based on the expression level of each gene in the gene expression matrix, thereby identifying the pathway enrichment of the different groups (Subramanian et al., 2005). The Molecular Signatures Database (MSigDB) gene set (c2.cp.kegg.v7.4.symbols.gmt; c5.go.v7.4.symbols.gmt) was selected to perform enrichment analysis, investigating the significantly enriched pathways between the two different risk groups. Statistical significance was set at *P*< 0.05 and false discovery rate (FDR) q < 0.25.

### The investigation of the immune status

Estimation of Stromal and Immune cells In Malignant Tumor tissues using Expression (ESTIMATE) is a method to infer the fraction of stromal cells and immune cells in tumors based on the expression of characteristic genes in transcriptome data and is widely used to assess tumor purity (Yoshihara et al., 2013). The R package “estimate” was utilized to assess the tumor immune infiltration score and tumor microenvironment score of OS. Then, the correlation and subgroup survival analysis was conducted between the risk scores and immune scores. Meantime, we downloaded the immune gene set from the MSigDB database and performed single-sample gene set enrichment analysis (ssGSEA) in OS to obtain the enrichment scores of various types of immune cells. Then, the differences in tumor immune cell infiltrating and immune function between the two risk groups were explored. Additionally, the CIBERSORT algorithm is an approach for characterizing the composition of immune cells in complex tissues based on gene expression data and is used to estimate the infiltrating immune cells in OS tissues (Gentles et al., 2015).

The related survival data is merged with the amount of immune cell infiltration to investigate the association between the novel prognostic signature and immune cell infiltration. Finally, we also compared the expression of immune checkpoints in the high- and low-risk group. The potential immune checkpoint was obtained from the previous report (Tang et al., 2021).

### Drug sensitivity analysis

Currently, the “pRRophetic” R package is commonly implemented for chemotherapeutic response prediction (Geeleher et al., 2014; Bray et al., 2018). To explore the difference in the anticancer drug sensitivity between the high-risk and low-risk groups, the R package “pRRophetic” was performed to calculate the half-maximal inhibitory concentration (IC50). Subsequently, the Wilcoxon signed-rank test was used to compare the differences in IC50 between the high-risk and low-risk groups.

### Cell culture

Human osteosarcoma cell lines 143B, U2OS, HOS, and normal osteoblast cell line hFOB1.19 were obtained from the American Type Culture Collection (ATCC). All the cell lines were cultured in Dulbecco’smodified Eagle’s medium (Gibco, United States) with 10% fetal bovine serum (Gibco, United States) and 1% penicillin-streptomycin solution (NCM Biotech, China) at 37°C with 5% CO_2_ in a humidified incubator.

### RT-qPCR

Total RNA was extracted with RNA Express Total RNA Kit (M050, NCM Biotech, China). Then, the Revert Aid First Strand cDNA Synthesis Kit (K1622, Thermo Scientific, United States) was used for cDNA synthesis. Subsequently, RT-qPCR was performed with Hieff qPCR SYBR Green Master Mix (High Rox Plus) (11203ES, YEASEN Biotech Co., Ltd., China). The primers used for the validation are listed in Supplementary Table S2.

### Statistical analysis

All statistical analyses in this study were conducted using R statistical software (Version 4.0.5). The Wilcox test analyzed differentially expressed necroptosis-related lncRNAs between osteosarcoma and normal tissues. K-M curve was performed for survival analyses, and the log-rank test was applied to analyze differences between subgroups. Univariate and multivariate Cox regression analyses were used to identify independent prognostic factors. The ROC curve was drawn to examine the sensitivity and specificity of the proposed predictive model for OS. The “Rtsne” R package was used to perform principal component analysis (PCA) and T-distributed stochastic neighbor embedding (t-SNE).

## Results

### Differentially expressed necroptosis-related lncRNAs

**FIGURE 1:**
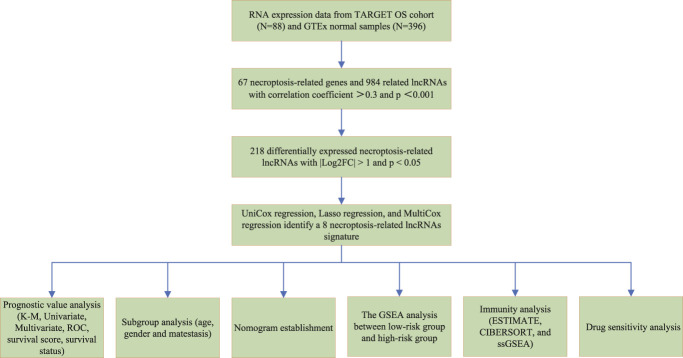
Flow chart of the study.

**FIGURE 2:**
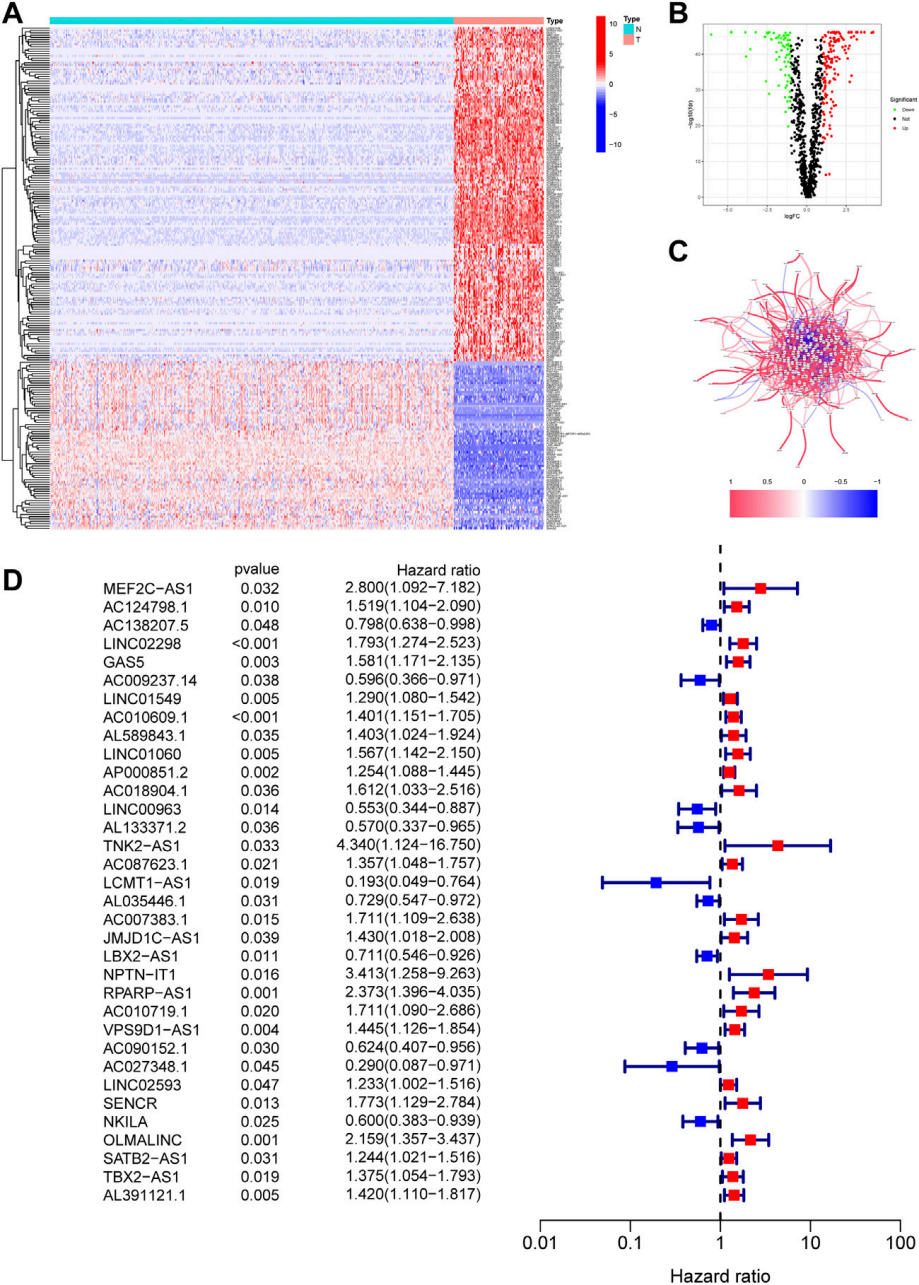
Identification of necroptosis-related lncRNAs in OS. **(A)**. The heatmap of 218 differentially expressed necroptosis-related lncRNAs. T represents tumor tissue, and N represents normal tissue. **(B)**. The volcano of 218 differentially expressed necroptosis-related lncRNAs. **(C)**. The correlation network between necroptosis-related lncRNAs. **(D)**. The prognostic lncRNAs extracted by univariate Cox regression analysis.

The flow diagram of our study is presented in Figure 1. First, we obtained 67 necroptosis-related genes from the previous literature, and Supplementary Table S3 displayed detailed information on these genes. Based on the co-expression relationship between lncRNAs and necroptosis-related genes, a total of 984 necroptosis-related lncRNAs were discovered (Supplementary Table S4). Subsequently, the differential analysis of the necroptosis-related lncRNAs between the 88 OS tissues and 396 normal tissues identified 218 differentially expressed necroptosis-related lncRNAs (Supplementary Table S5). Among these, 145 genes were up-regulation while 73 genes were down-regulation. Figures 2A,B shows the heatmaps and volcano plots of differentially expressed necroptosis-related lncRNAs, respectively. The correlation network between necroptosis-related lncRNAs is shown in Figure 2C.

### Establishment of necroptosis-related lncRNAs prognostic signature of OS

**FIGURE 3:**
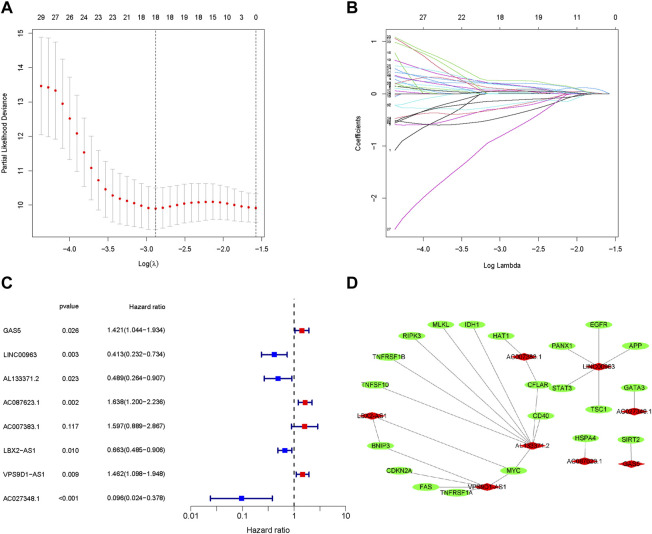
Construction of a prognostic pyroptosis-associated lncRNA signature. **(A,B)**. The LASSO Cox analysis determined 15 prognostic genes. **(C)**. The forest map of multivariate Cox analysis. **(D)**. The network between lncRNAs and necroptosis genes (correlation coefficients > 0.3 and *p* < 0.001).

The 218 differentially expressed necroptosis-related lncRNAs were used for subsequent prognostic model construction. First, we performed a univariate Cox regression analysis to identify necroptosis-related lncRNAs connected with OS prognosis. The results discovered that 34 differentially expressed necroptosis-related lncRNAs were closely connected with the overall survival of patients with OS (Figure 2D). Then, the LASSO regression analysis of these 34 lncRNAs showed that the regression parameter λ value was best when selected 18 lncRNAs (Figures 3A,B). To further optimize the prognostic signature, we conducted a multivariate COX regression analysis on these 18 lncRNAs. Thus, a prognostic model including eight differentially expressed necroptosis-related lncRNAs was constructed according to the multivariate Cox regression (Figure 3C and Supplementary Table S6). The network between these eight lncRNAs and necroptosis-related genes was presented in Figure 3D. Finally, the formula of risk score was as follows: Risk score = 0.351230254536139 *GAS5 + (-0.885405813420193)*LINC00963 + (-0.71485613341702)*AL133371.2 + 0.493281991774*AC087623.1 + 0.467979376327946*AC007383.1 + (-0.410460487658081)*LBX2-AS1 + 0.380008102384851*VPS9D1-AS1 + (-2.34811257258056)*AC027348.1. The risk score of each OS patient was calculated using the algorithm and split into high- and low-risk groups based on the median risk score for further analysis. Supplementary Figure S1 demonstrated the differences in risk scores between different clinical groups.

### Assessment of the novel risk signature

**FIGURE 4:**
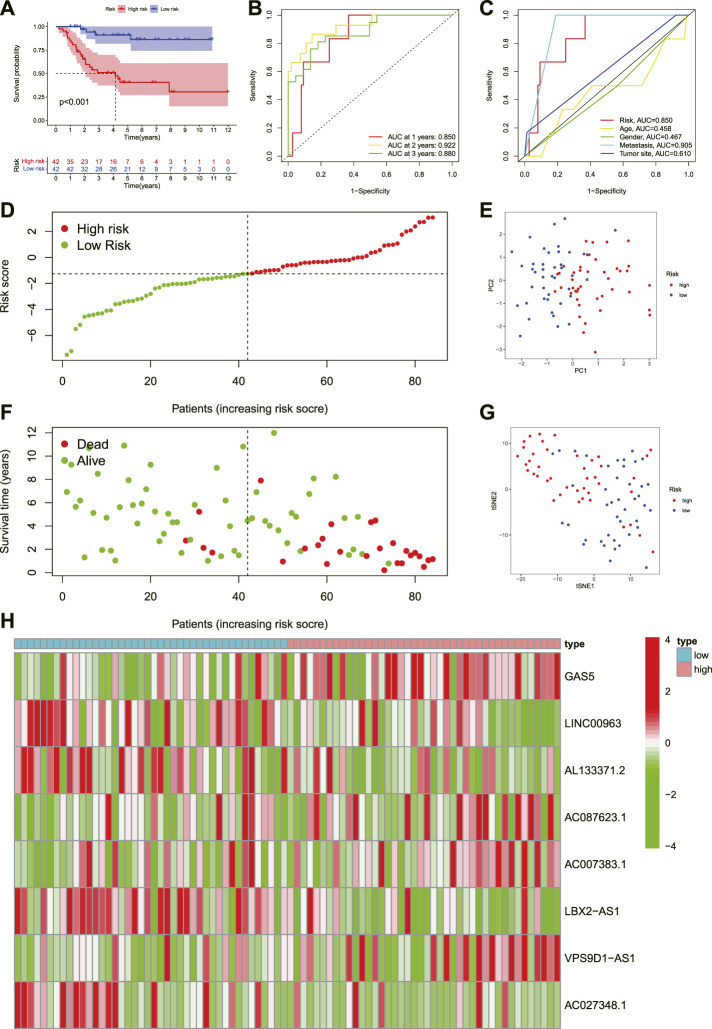
Prognosis value of the eight necroptosis-related lncRNAs signature. **(A)**. The outcome of the Kaplan-Meier curves. **(B)**. The AUC values for predicting OS survival rates at 1, 2, and 3 years. **(C)**. The AUC values of the risk variables. **(D)**. Distribution of patient risk ratings. **(E)**. The PCA plot. **(F)**. Plot of risk survival status. **(G)**. The t-SNE plot. **(H)**. Heatmap of lncRNAs associated with necroptosis in high- and low-risk groups.

To understand the prognostic model’s predictive value, we conducted the K-M survival analysis to compare the difference in survival status between the high and low-risk groups. We found that the prognosis of OS in the low-risk group was better than that of the high-risk group (Figure 4A, *p* < 0.001 ). Similarly, the risk score curve and survival status plots indicated that the OS patients in the low-risk group had a better prognosis, with fewer deaths and longer survival time (Figure 4D–F). Moreover, the PCA and t-SNE suggested that the OS patients in different groups were distributed in two directions (Figure 4E,G). Subsequently, we established a ROC curve to assess the prognostic prediction efficiency of this novel necroptosis-related lncRNAs signature. The result illustrated that AUC was 0.850 (1-year overall survival), 0.922 (2- years overall survival), and 0.88 (3-years overall survival) (Figure 4B). Meanwhile, the ROC curve of clinical factors and risk score show metastasis, and the novel signature has a predominant predictive ability (Figure 4C,H). shows the risk heat map of this necroptosis-related lncRNAs signature. In general, these results implied that the novel necroptosis-related lncRNA signature was well established and had an excellent predictive ability.

### Independent prognostic value of the novel prognostic signature

**FIGURE 5:**
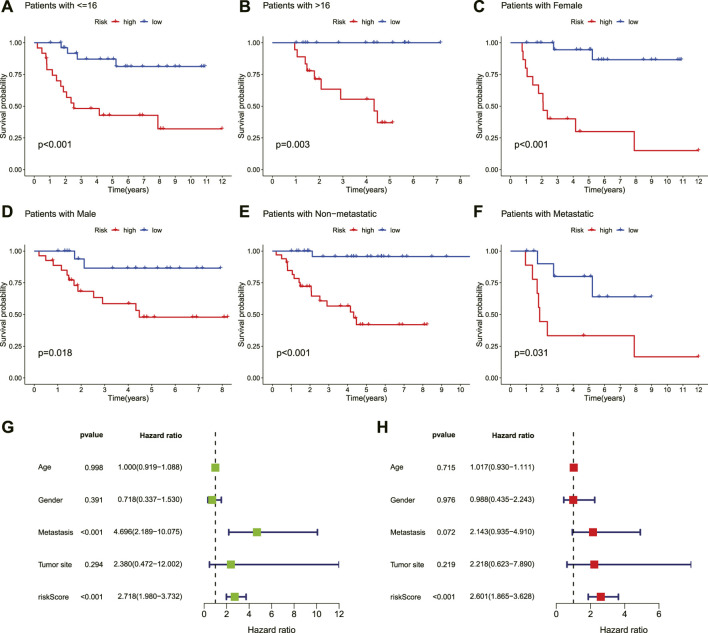
Independent prognostic value of the novel prognostic signature. **(A–F)**. Kaplan–Meier survival curves are stratified by age, gender, and metastasis status. **(G)**. Univariate analysis result. **(H)**. Multivariate analysis result.

To evaluate whether the novel necroptosis-related lncRNA signature was independent of other clinical factors, we categorized patients with OS into different clinical subgroups based on their age (≤16, >16), gender, and metastasis status and analyzed the survival differences between the high- and low-risk groups in different subgroups. The results indicated that OS patients in the low-risk group had a better clinical prognosis than the high-risk group among different ages, genders, and metastatic status (Figures 5A–F). Moreover, the univariate COX analysis identified metastasis status and risk score as overall survival-related variables (Figure 5G). Multivariate analyses also revealed that the risk score was an independent risk factor affecting the prognosis of OS (Figure 5F). Hence, these findings illustrated that the novel necroptosis-related signature is an independent predicted factor and does not correlate with other clinical factors.

### Construction of nomogram

**FIGURE 6:**
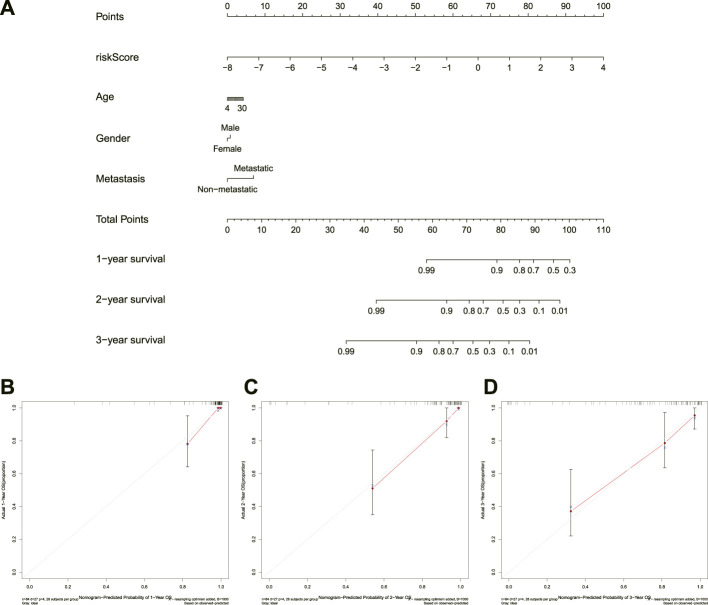
Construction and calibration of a nomogram. **(A)**. Nomogram integrating risk score and clinical characteristics. **(B–D)**, calibration of the nomogram at 1-year, 2-years, and 3-years survival.

Incorporating the risk score and clinical parameters, we constructed a nomogram for predicting 1-, 2-, and 3-years overall survival. As shown in Figure 6A, this nomogram can predict patient outcomes individually based on the various patient conditions. Additionally, the calibration curve was utilized to predict the accuracy of the nomogram, and the results revealed that the nomogram had an accordant agreement with the prediction of 1-, 2-, and 3-years overall survival (Figures 6B–D). Collectively, the novel necroptosis-related lncRNAs prognostic signature was found to be stable and accurate, suggesting that it might be used in the therapeutic therapy of OS.

### GSEA results

**FIGURE 7:**
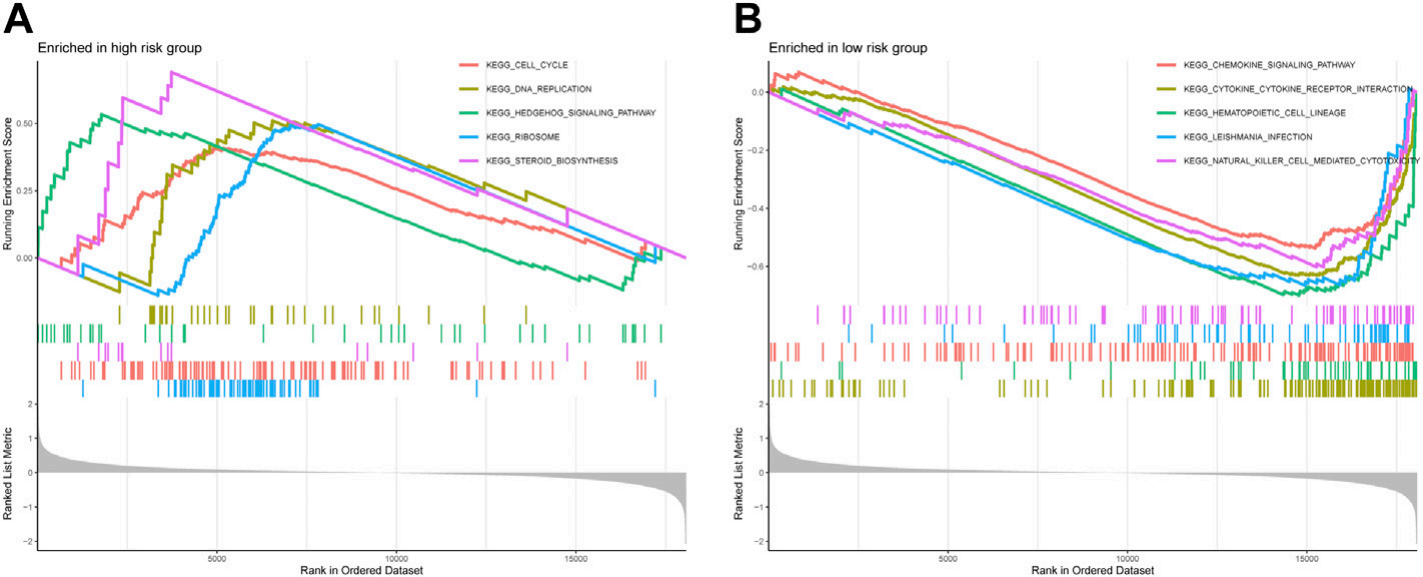
The GSEA analysis results between the high-risk group and low-risk group. **(A)**. Top five enriched pathways in the high-risk group. **(B)**. Top five enriched pathways in the low-risk group.

The GSEA was performed to investigate the potential process and pathway involved in molecular heterogeneity between the two risk groups. Supplementary Table S7 showed all 45 significantly enriched pathways, and Figure 7 presented the top five enriched pathways in the high- and low-risk group. As shown in the outcomes, we found that the high-risk groups enriched in several tumor development-related pathways, such as the Cell cycle, the Hedgehog signaling pathway, and DNA replication. On the other hand, we knew that most of the immune-related pathways were enriched in the low-risk group, such as Cytokine-cytokine receptor interaction, Primary immunodeficiency, Natural killer cell mediated cytotoxicity, Intestinal immune network for IgA production, Autoimmune thyroid disease, Systemic lupus erythematosus, T/B cell receptor signaling pathway, Toll-like receptor (TLR) signaling pathway, and Antigen processing and presentation. Overall, the above results illustrated that the novel necroptosis-related lncRNAs signature regulated these tumor and immune-related pathways, which may significantly affect the tumorigenesis of OS. Therefore, we tried to perform an immune analysis on the signature.

### The immune status of different risk groups

**FIGURE 8:**
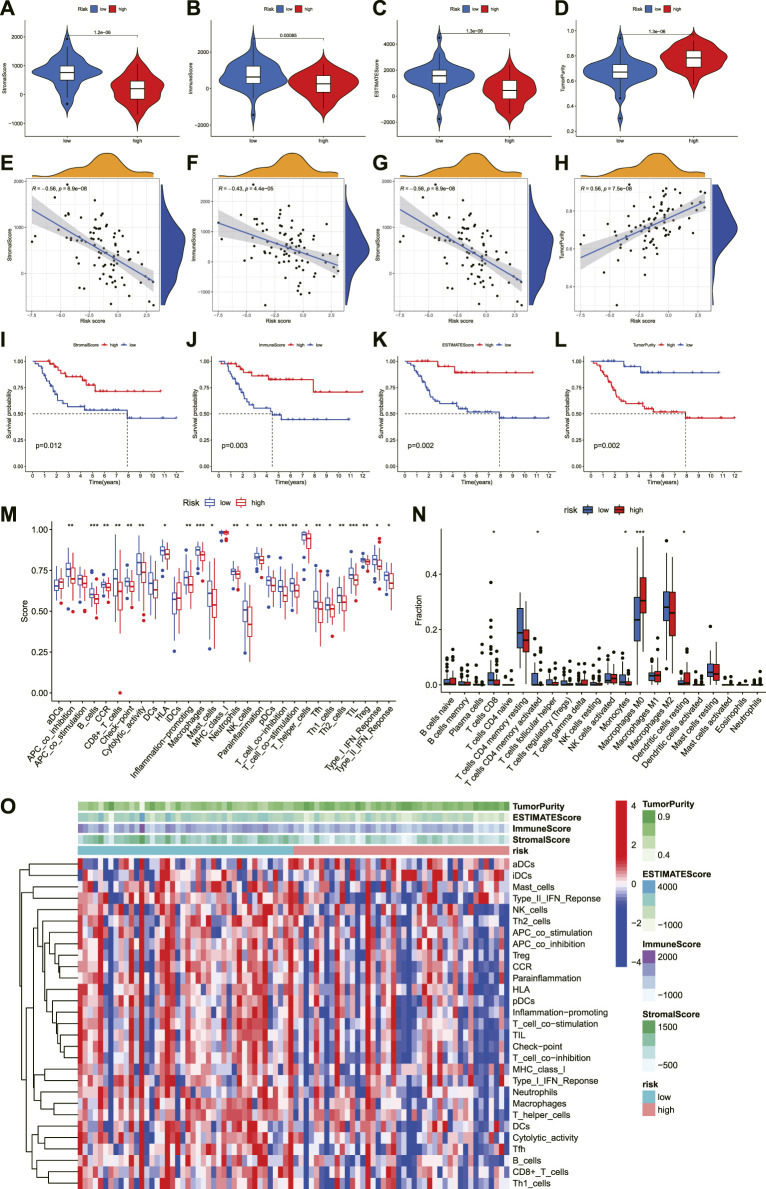
The investigation of immune status in OS. **(A–D)**. Comparisons between the two risk groups regarding stomal score, immune score, ESTIMATE score, and tumor purity. **(E–H)**. The correlation between risk score and stomal score, immune score, ESTIMATE score, and tumor purity. **(I–L)**. **(K–M)** survival outcome is based on different stomal scores, immune scores, ESTIMATE scores, and tumor purity. **(M)**. The boxplot of tumor-infiltrating immune cell types in low- and high-risk groups. **(N)**. Boxplots of immune cells and immune-associated functions in low- and high-risk groups. **(O)**. Heatmap for immune status based ESTIMATE and ssGSEA among two risk subgroups.

To explore the roles of necroptosis-related lncRNAs signature in the tumor immune microenvironment, we further analyzed the association between the immune status and the risk score in OS. First, we calculated the immune score of OS through ESTIMATE and compared its correlation with the risk score and survival prognosis. The immune, stromal, and estimation scores were all greater in the low-risk group than in the high-risk group, but tumor purity was lower in the low-risk group than in the high-risk group (Figures 8A–H). Similarly, immune score, stromal score, and estimate score positively correlate with survival time, whereas tumor purity is negatively correlated with survival time (Figure 8I–L). These results reveal that the immune infiltration and survival time decreased with the increase in risk score. Meanwhile, the ssGSEA indicated that the proportion of almost all immune cells, the component level, and related pathway function significantly increased in the low-risk subgroup (Figure 8M). Only the scores of aDCs, APC_co_stimulation, DCs, iDCs, and MHC_class_I was no significant difference between the two risk groups (*p* > 0.05). This supported the observation that the degree of immune infiltration in the low-risk group was higher than in the high-risk group.

In addition to analyzing the correspondence scores of the immune microenvironment, it is also necessary to understand the proportion of the immune landscape of OS. We performed the CIBERSORT algorithm to explore the proportion of immune cells in OS. As shown in Figure 8N, the proportions of T cells CD8, T cells CD4 memory activated, Monocytes, Macrophages M0, and Dendritic cells resting were statistically significant between the low- and high-risk score groups. According to the correlation analysis, the risk score was also highly connected with several immune cells. The result illustrated that the risk score was negatively correlated with T cells CD8, T cells CD4 memory activated, and Monocytes, while positively correlated with Macrophages M0 (Supplementary Figure S2). Moreover, the infiltration of these immune cells was associated with the prognosis of OS patients (Supplementary Figure S3). Finally, we compared the expression level of immune checkpoints between the high- and low-risk groups. The results revealed that most immune checkpoints showed better activation in the low-risk group (Supplementary Figure S4). Hence, these results proved that the novel necroptosis-related lncRNA signature was closely related to the immune microenvironment of OS, and necroptosis-related lncRNAs may affect the prognosis of OS patients by regulating the immune status.

### Correlation between necroptosis-related lncRNAs signature gene and drug sensitivity

**FIGURE 9:**
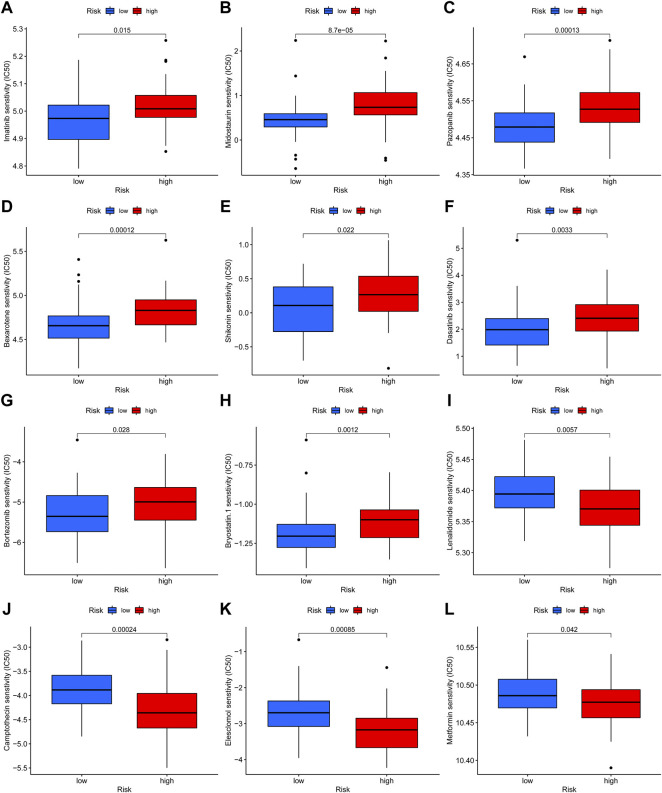
Drug sensitivity analysis. **(A–L)**. The candidate anticancer drugs with significant treatment differences in the high- and low-risk groups.

Additionally, we compared the susceptibility of OS to commonly used anticancer drugs between the two distinct risk groups to identify potential OS treatment agents. We discovered that the OS patients in the low-risk group might positively react to Imatinib, Midostaurin, Pazopanib, Bexarotene, Shikonin, Dasatinib, Bortezomib, and Bryostatin, while patients with high-risk scores maybe respond better to Lenalidomide, Camptothecin, Elesclomol, and Metformin (Figure 9). Under these circumstances, the novel necroptosis-related lncRNAs signature may be utilized to guide chemotherapy selection of OS in the future.

### Validation of the several necroptosis-related lncRNAs in OS

**FIGURE 10:**
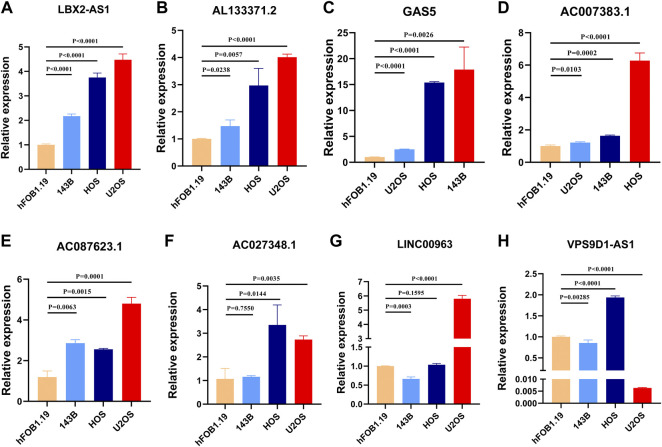
**(A–H)**. Validation of the expression of signature genes in OS cell lines. **(A)**. LBX2-AS1. **(B)**. AL133371.2. **(C)**. GAS5. **(D)**. AC007383.1. **(E)**. AC087623.1. **(F)**. AC027348.1. **(G)**. LINC00963. **(H)**. VPS9D1-AS1.

The expression level of these signature necroptosis-related lncRNAs in cell lines (human OS cell line: 143B, HOS, and U2OS and normal osteoclast cell lines: hFOB1.19) were further detected through RT-qPCR. As presented in Figures 10A–E, the lncRNA LBX2-AS1, AL133371.2, GAS5, AC007383.1, and AC087623.1 were elevated in OS cell lines compared to normal cell lines. Meanwhile, the lncRNA AC0273484.1 exhibited an augmented expression in HOS and U2OS cell lines (Figure 10F). Additionally, the LINC00963 and VPS9D1-AS1 displayed a different expression in OS cell lines (Figures 10G–H). For LINC00963, it is overexpressed in U2OS while downregulated in 143B. The lncRNA VPS9D1-AS1 had an upregulated expression level in HOS and a diminished expression in 143B and U2OS.

## Discussion

OS is the most common bone malignancy in children and adolescents (Niu et al., 2020). Although standard multimodal therapies (surgery, radiotherapy, chemotherapy, and immunotherapy) have significantly improved survival rates, some patients' prognoses with OS continue to be poor (Durfee et al., 2016). The characteristics of drug resistance and early metastasis of OS have led to a greatly reduced treatment effect, and the progress of OS treatment seems to reach an apparent plateau (Hameed and Dorfman, 2011). Therefore, finding effective prognostic markers is essential for diagnosis and early active intervention of OS. Recently, as a new type of programmed cell death, necroptosis has been discovered that command a dual role in the development and treatment of cancer. On the other hand, promoting necroptosis could be a potential therapeutic target for tumors (Yan et al., 2022). In addition, increasing evidence showed that the lncRNA was associated with tumor progression. Several novel lncRNA models based on autophagy, pyroptosis, and inflammation-related genes have also been used to predict the prognosis of tumor patients (Wang et al., 2019; Ping et al., 2021; Xiang et al., 2021). Furthermore, studies have revealed that the necroptosis-related lncRNA model can precisely predict the prognosis of gastric cancer and help to guide individualized treatment for gastric cancer (Zhao et al., 2021). However, there are no reports investigating the association between necroptosis-related lncRNA and the clinical characteristics of patients with OS. Therefore, in this study, we constructed a comprehensive prognostic signature based on necroptosis-related lncRNA and explored the potential molecular mechanisms and signal transduction of OS progression. To the best of our knowledge, this is the first comprehensive investigation that explored the roles of necroptosis-related lncRNA in OS.

A total of 67 necroptosis-related genes were obtained from previous studies, and 218 differentially expressed necroptosis-related lncRNAs were identified through Pearson correlation analysis and difference analysis. Then, we screened eight necroptosis-related lncRNAs for the subsequent signature establishment based on univariate Cox regression analysis, Lasso regression, and multivariate Cox regression analysis. Among these prognostic necroptosis-related lncRNAs in OS, some of them have been demonstrated to play pivotal roles in carcinogenesis and progression. For instance, the lncRNA VPS9D1-AS1 was significantly upregulated in esophageal squamous cell carcinoma, which can promote the malignant progression of esophageal squamous cell carcinoma through the Wnt/catenin signaling pathway (Ma et al., 2021). In colon adenocarcinoma, VPS9D1-AS1 can upregulate CLDN1 by sponging miR-1301-3p, thus facilitating colon adenocarcinoma cell growth suppressing apoptosis (Liu, 2021). More importantly, we preliminarily measured the expression of these signature lncRNAs in OS cell lines and osteoclast cells. These results give a novel insight into the future biomarker exploration in OS. However, only a few lncRNAs related to necroptosis have been reported, and a comprehensive analysis is needed to clarify their role in OS.

To systematically analyze the prognostic accuracy of necroptosis-related lncRNAs in OS, we conducted a novel prognosis signature based on these eight necroptosis-related lncRNAs. The patients were split into two risk groups according to the risk scores, and various bioinformatics analyses were performed. First, our survival analysis results indicated that the survival prognosis of patients in the high-risk group is significantly lower than that in the low-risk group. The survival analysis results between different clinical subgroups are also consistent with that. Meanwhile, the 1-year, 2-years, and 3-years ROC curves confirmed the accuracy of the novel signature in survival prediction. Additionally, univariate and multivariate analyses further verified that the risk model could be an independent prognostic factor for OS. Finally, the necroptosis-related prognostic nomogram and calibration curve demonstrated that the signature could accurately predict OS patient’s prognosis. Consequently, these above results revealed that the novel prognostic risk signature based on these eight necroptosis-related lncRNAs has better predictive performance and universal applicability.

After that, to further explore the association between the novel necroptosis-related lncRNAs signature and OS GSEA was utilized, and the results revealed that the high-risk group significantly enriched several cancer-related signaling pathways, including DNA replication, Hedgehog signaling pathway, and cell cycle. Accumulated evidence has demonstrated a vital role for these signaling pathways in the development and progression of OS. To name a few, microRNA-524-5p inhibits the proliferation of OS cells and induces cell cycle arrest by targeting CD6 (Chen H. et al., 2020). URG4, which is overexpressed in OS, promotes cell cycle progression based on activating the GSK3β/β-catenin/cyclin D1 signaling pathway (Liu et al., 2020). Similarly, the Hedgehog signaling pathway has been confirmed to have a significant role in OS. Dong-Dong Cheng et al. proved that CNOT1 and LMNA cooperate in aggravating the tumorigenesis of OS *via* the Hedgehog signaling pathway (Cheng et al., 2017). On the contrary, the low-risk OS patients were significantly enriched in immunological pathways, such as Cytokine-cytokine receptor interaction, Natural killer cell mediated cytotoxicity, T/B cell receptor signaling pathway, Toll-like receptor signaling pathway, and antigen processing and presentation. It was consistent with numerous prior research results (Bu et al., 2021; Ping et al., 2021; Tang et al., 2021). For instance, Xinxin Bu et al. revealed that the pyroptosis-related lncRNA signature was mainly enriched in the pathway associated with the immune process (Bu et al., 2021). The novel ferroptosis-related lncRNAs prognostic signature for Head and neck squamous cell carcinoma regulated most immune-related pathways (Tang et al., 2021). Additionally, these immune-related signal pathways are solid factors for tumorigenesis and progression (Gentles et al., 2015). For example, the involvement and activation of TLRs and the TLR signaling pathway can enhance the malignant phenotype of bladder tumors and finally lead to the progress of the tumor (Basith et al., 2012). Together, it is reasonable to speculate that necroptosis-related lncRNAs signature affecting the prognosis of OS patients may be attributed to their relationship with immune status.

To further explore the relationship between the necroptosis-related lncRNAs signature and the immune status of osteosarcoma, we performed ESTIMATE, ssGSEA, and CIBERSORT algorithms to analyze the immune infiltration status and the tumor microenvironment in O.S. First, the ESTIMATE analysis indicated that the overall immune score of OS was negatively correlated with the risk score and was correlated with the prognosis of patients with OS Similarly, the ssGSEA showed that most of the immune cell infiltration and corresponding immune functions in the low-risk group were better than those in the high-risk group. This was in line with the results of several previous research. Xinxin Bu et al. showed a lower immune infiltration score in the high-risk group of pyroptosis-related lncRNAs signature for OS (Bu et al., 2021). Ting Lei et al. revealed that the ferroptosis-related gene signature could affect the immune state of the tumor microenvironment, thereby regulating the progression and prognosis of OS (Lei et al., 2021). In addition, we also found different levels of several immune cell infiltration between the different risk groups through the CIBERSORT algorithm. The risk score was negatively correlated with the degree of infiltration of T cells CD8, T cells CD4 memory activated, and Monocytes, while positively correlated with Macrophages M0, and these immune cells are linked to the prognoses of patients with OS. Meanwhile, considerable evidence proved that immune cell infiltration was significantly related to the prognosis of OS. For instance, CD8^+^ T cells play a critical role in anti-tumor immunity. High CD8^+^ T cell infiltration levels are significantly related to the better prognosis of various cancers, including OS (Nowicki et al., 2017; Tang et al., 2019; Zhu and Hou, 2020). Previous studies have demonstrated that a higher estimated fraction of M0 macrophages was associated with a poor prognosis of sarcoma (Zhu and Hou, 2020; Fan et al., 2021). Finally, we also revealed that the expression level of several immune checkpoints was negatively connected with the risk score. Cancer immunotherapy targeting immune checkpoints has improved the prognosis of various cancer patients (Iwai et al., 2017). Hence, the results of our study showed that necroptosis-related lncRNAs signature affects the prognosis of OS by regulating the immune status.

Subsequently, we determined the drug sensitivity to candidate anticancer drugs in the two risk groups. Our results revealed that Imatinib, Midostaurin, Pazopanib, Bexarotene, Shikonin, Dasatinib, Bortezomib, and Bryostatin showed better responses in the low-risk score group. In contrast, Lenalidomide, Camptothecin, Elesclomol, and Metformin in the high-risk group showed better response. In the past, it has been shown that the application of imatinib and adriamycin had a synergistic antiproliferative effect in platelet-derived growth factor receptor-expressing OS cells (Yamaguchi et al., 2015). Shikonin, an effective constituent extracted from a Chinese medicinal herb, was demonstrated to inhibit the progress of OS (Fu et al., 2013; Deng and Qiu, 2015). For instance, Biyong Deng et al. revealed that Shikonin could inhibit the invasiveness of OS *via* MMP13 inhibition (Deng and Qiu, 2015). Equally, Metformin can induce the cell cycle arrest, apoptosis, and autophagy of OS through ROS/JNK signaling pathway (Li B. et al., 2020). Overall, the present study implies that the novel necroptosis-related lncRNA signature may help to screen chemotherapeutic agents for individualized treatment in OS.

## Conclusion

In summary, our study constructed a novel necroptosis-related lncRNAs signature, which could effectively predict the prognosis of OS. The signature could be served as an independent prognostic factor for OS. Furthermore, the GSEA and immune analyses revealed that these necroptosis-related lncRNAs affect the OS prognosis *via* regulating the immune status. It is reasonable to believe that our study provides new insight into prognosis prediction and individualized chemotherapy therapy for OS.

## Data Availability

Publicly available datasets were analyzed in this study. This data can be found here: TCGA (https://portal.gdc.cancer.gov/), and GTEx (https://gtexportal.org/).

## References

[B1] Amaravadi R. K. Kimmelman A. C. Debnath J. (2019). Targeting autophagy in cancer: Recent advances and future directions. Cancer Discov. 9 (9), 1167–1181. 10.1158/2159-8290.Cd-19-0292 31434711 PMC7306856

[B2] Basith S. Manavalan B. Yoo T. H. Kim S. G. Choi S. (2012). Roles of toll-like receptors in cancer: A double-edged sword for defense and offense. Arch. Pharm. Res. 35 (8), 1297–1316. 10.1007/s12272-012-0802-7 22941474

[B3] Bray F. Ferlay J. Soerjomataram I. Siegel R. L. Torre L. A. Jemal A. (2018). Global cancer statistics 2018: GLOBOCAN estimates of incidence and mortality worldwide for 36 cancers in 185 countries. Ca. Cancer J. Clin. 68 (6), 394–424. 10.3322/caac.21492 30207593

[B4] Bu X. Liu J. Ding R. Li Z. (2021). Prognostic value of a pyroptosis-related long noncoding RNA signature associated with osteosarcoma microenvironment. J. Oncol. 2021, 2182761. 10.1155/2021/2182761 34804157 PMC8601829

[B5] Chen H. Cheng C. Gao S. (2020a). microRNA-524-5p inhibits proliferation and induces cell cycle arrest of osteosarcoma cells *via* targeting CDK6. Biochem. Biophys. Res. Commun. 530 (3), 566–573. 10.1016/j.bbrc.2020.07.092 32747087

[B6] Chen Z. Chen Q. Cheng Z. Gu J. Feng W. Lei T. (2020b). Long non-coding RNA CASC9 promotes gefitinib resistance in NSCLC by epigenetic repression of DUSP1. Cell Death Dis. 11 (10), 858. 10.1038/s41419-020-03047-y 33056982 PMC7560854

[B7] Cheng D. D. Li J. Li S. J. Yang Q. C. Fan C. Y. (2017). CNOT1 cooperates with LMNA to aggravate osteosarcoma tumorigenesis through the Hedgehog signaling pathway. Mol. Oncol. 11 (4), 388–404. 10.1002/1878-0261.12043 28188704 PMC5527480

[B8] Consortium T. G. (2020). The GTEx Consortium atlas of genetic regulatory effects across human tissues. Science 369, 1318–1330. 10.1126/science.aaz1776 32913098 PMC7737656

[B9] Deng B. Qiu B. (2015). Shikonin inhibits invasiveness of osteosarcoma through MMP13 suppression. Tumour Biol. 36 (12), 9311–9317. 10.1007/s13277-015-3662-1 26104765

[B10] Durfee R. A. Mohammed M. Luu H. H. (2016). Review of osteosarcoma and current management. Rheumatol. Ther. 3 (2), 221–243. 10.1007/s40744-016-0046-y 27761754 PMC5127970

[B11] Fan J. Qin X. He R. Ma J. Wei Q. (2021). Gene expression profiles for an immunoscore model in bone and soft tissue sarcoma. Aging (Albany NY) 13 (10), 13708–13725. 10.18632/aging.202956 33946044 PMC8202872

[B12] Fu Z. Deng B. Liao Y. Shan L. Yin F. Wang Z. (2013). The anti-tumor effect of shikonin on osteosarcoma by inducing RIP1 and RIP3 dependent necroptosis. BMC Cancer 13, 580. 10.1186/1471-2407-13-580 24314238 PMC4028842

[B13] Galluzzi L. Vitale I. Aaronson S. A. Abrams J. M. Adam D. Agostinis P. (2018). Molecular mechanisms of cell death: Recommendations of the nomenclature committee on cell death 2018. Cell Death Differ. 25 (3), 486–541. 10.1038/s41418-017-0012-4 29362479 PMC5864239

[B14] Geeleher P. Cox N. Huang R. S. (2014). pRRophetic: an R package for prediction of clinical chemotherapeutic response from tumor gene expression levels. PLoS One 9 (9), e107468. 10.1371/journal.pone.0107468 25229481 PMC4167990

[B15] Gentles A. J. Newman A. M. Liu C. L. Bratman S. V. Feng W. Kim D. (2015). The prognostic landscape of genes and infiltrating immune cells across human cancers. Nat. Med. 21 (8), 938–945. 10.1038/nm.3909 26193342 PMC4852857

[B16] Goldman M. Craft B. Swatloski T. Cline M. Morozova O. Diekhans M. (2015). The UCSC cancer genomics browser: Update 2015. Nucleic Acids Res. 43, D812–D817. (Database issue). 10.1093/nar/gku1073 25392408 PMC4383911

[B17] Goričar K. Kovač V. Jazbec J. Zakotnik B. Lamovec J. Dolžan V. (2014). Influence of the folate pathway and transporter polymorphisms on methotrexate treatment outcome in osteosarcoma. Pharmacogenet. Genomics 24 (10), 514–521. 10.1097/fpc.0000000000000083 25098908

[B18] Grootjans S. Vanden Berghe T. Vandenabeele P. (2017). Initiation and execution mechanisms of necroptosis: An overview. Cell Death Differ. 24 (7), 1184–1195. 10.1038/cdd.2017.65 28498367 PMC5520172

[B19] Hameed M. Dorfman H. (2011). Primary malignant bone tumors--recent developments. Semin. Diagn. Pathol. 28 (1), 86–101. 10.1053/j.semdp.2011.02.002 21675380

[B20] Harrison D. J. Geller D. S. Gill J. D. Lewis V. O. Gorlick R. (2018). Current and future therapeutic approaches for osteosarcoma. Expert Rev. Anticancer Ther. 18 (1), 39–50. 10.1080/14737140.2018.1413939 29210294

[B21] Iwai Y. Hamanishi J. Chamoto K. Honjo T. (2017). Cancer immunotherapies targeting the PD-1 signaling pathway. J. Biomed. Sci. 24 (1), 26. 10.1186/s12929-017-0329-9 28376884 PMC5381059

[B22] Jiang N. Zhang X. Gu X. Li X. Shang L. (2021). Progress in understanding the role of lncRNA in programmed cell death. Cell Death Discov. 7 (1), 30. 10.1038/s41420-021-00407-1 33558499 PMC7870930

[B23] Kempf-Bielack B. Bielack S. S. Jürgens H. Branscheid D. Berdel W. E. Exner G. U. (2005). Osteosarcoma relapse after combined modality therapy: An analysis of unselected patients in the cooperative osteosarcoma study group (COSS). J. Clin. Oncol. 23 (3), 559–568. 10.1200/jco.2005.04.063 15659502

[B24] Lei T. Qian H. Lei P. Hu Y. (2021). Ferroptosis-related gene signature associates with immunity and predicts prognosis accurately in patients with osteosarcoma. Cancer Sci. 112 (11), 4785–4798. 10.1111/cas.15131 34506683 PMC8586685

[B25] Li B. Zhou P. Xu K. Chen T. Jiao J. Wei H. (2020a). Metformin induces cell cycle arrest, apoptosis and autophagy through ROS/JNK signaling pathway in human osteosarcoma. Int. J. Biol. Sci. 16 (1), 74–84. 10.7150/ijbs.33787 31892847 PMC6930379

[B26] Li X. Li Y. Yu X. Jin F. (2020b). Identification and validation of stemness-related lncRNA prognostic signature for breast cancer. J. Transl. Med. 18 (1), 331. 10.1186/s12967-020-02497-4 32867770 PMC7461324

[B27] Liu W. (2021). Long non-coding RNA VPS9D1-AS1 promotes growth of colon adenocarcinoma by sponging miR-1301-3p and CLDN1. Hum. Cell 34 (6), 1775–1787. 10.1007/s13577-021-00604-1 34519940

[B28] Liu Y. Xi Y. Chen G. Wu X. He M. (2020). URG4 mediates cell proliferation and cell cycle in osteosarcoma *via* GSK3β/β-catenin/cyclin D1 signaling pathway. J. Orthop. Surg. Res. 15 (1), 226. 10.1186/s13018-020-01681-y 32552851 PMC7301506

[B29] Ma L. Yan W. Sun X. Chen P. (2021). Long non-coding RNA VPS9D1-AS1 promotes esophageal squamous cell carcinoma progression *via* the Wnt/β-catenin signaling pathway. J. Cancer 12 (22), 6894–6904. 10.7150/jca.54556 34659577 PMC8517997

[B30] Marshall K. D. Baines C. P. (2014). Necroptosis: Is there a role for mitochondria? Front. Physiol. 5, 323. 10.3389/fphys.2014.00323 25206339 PMC4144201

[B31] Niu J. Yan T. Guo W. Wang W. Zhao Z. Ren T. (2020). Identification of potential therapeutic targets and immune cell infiltration characteristics in osteosarcoma using bioinformatics strategy. Front. Oncol. 10, 1628. 10.3389/fonc.2020.01628 32974202 PMC7471873

[B32] Nowicki T. S. Akiyama R. Huang R. R. Shintaku I. P. Wang X. Tumeh P. C. (2017). Infiltration of CD8 T cells and expression of PD-1 and PD-L1 in synovial sarcoma. Cancer Immunol. Res. 5 (2), 118–126. 10.1158/2326-6066.Cir-16-0148 28039162 PMC5290092

[B33] Ohno T. Abe M. Tateishi A. Kako K. Miki H. (1975). Osteogenic sarcoma. A study of one hundred and thirty cases. J. Bone Jt. Surg. 57 (3), 397–404. 10.2106/00004623-197557030-00019 47330

[B34] Ping L. Zhang K. Ou X. Qiu X. Xiao X. (2021). A novel pyroptosis-associated long non-coding RNA signature predicts prognosis and tumor immune microenvironment of patients with breast cancer. Front. Cell Dev. Biol. 9, 727183. 10.3389/fcell.2021.727183 34616734 PMC8488148

[B35] Qin F. Tang H. Zhang Y. Zhang Z. Huang P. Zhu J. (2020). Bone marrow-derived mesenchymal stem cell-derived exosomal microRNA-208a promotes osteosarcoma cell proliferation, migration, and invasion. J. Cell. Physiol. 235 (5), 4734–4745. 10.1002/jcp.29351 31637737

[B36] Quinn J. J. Chang H. Y. (2016). Unique features of long non-coding RNA biogenesis and function. Nat. Rev. Genet. 17 (1), 47–62. 10.1038/nrg.2015.10 26666209

[B37] Rodriguez R. Rubio R. Menendez P. (2012). Modeling sarcomagenesis using multipotent mesenchymal stem cells. Cell Res. 22 (1), 62–77. 10.1038/cr.2011.157 21931359 PMC3351912

[B38] Sadykova L. R. Ntekim A. I. Muyangwa-Semenova M. Rutland C. S. Jeyapalan J. N. Blatt N. (2020). Epidemiology and risk factors of osteosarcoma. Cancer Invest.. 38 (5), 259–269. 10.1080/07357907.2020.1768401 32400205

[B39] Subramanian A. Tamayo P. Mootha V. K. Mukherjee S. Ebert B. L. Gillette M. A. (2005). Gene set enrichment analysis: A knowledge-based approach for interpreting genome-wide expression profiles. Proc. Natl. Acad. Sci. U. S. A. 102 (43), 15545–15550. 10.1073/pnas.0506580102 16199517 PMC1239896

[B40] Tang Y. Gu Z. Fu Y. Wang J. (2019). CXCR3 from chemokine receptor family correlates with immune infiltration and predicts poor survival in osteosarcoma. Biosci. Rep. 39 (11), BSR20192134. 10.1042/bsr20192134 31696204 PMC6851512

[B41] Tang Y. Li C. Zhang Y. J. Wu Z. H. (2021). Ferroptosis-Related Long Non-Coding RNA signature predicts the prognosis of Head and neck squamous cell carcinoma. Int. J. Biol. Sci. 17 (3), 702–711. 10.7150/ijbs.55552 33767582 PMC7975700

[B42] Tao J. Jiang M. M. Jiang L. Salvo J. S. Zeng H. C. Dawson B. (2014). Notch activation as a driver of osteogenic sarcoma. Cancer Cell 26 (3), 390–401. 10.1016/j.ccr.2014.07.023 25203324 PMC4159617

[B43] Tonnus W. Meyer C. Paliege A. Belavgeni A. von Mässenhausen A. Bornstein S. R. (2019). The pathological features of regulated necrosis. J. Pathol. 247 (5), 697–707. 10.1002/path.5248 30714148

[B44] Wang Z. Gao L. Guo X. Feng C. Lian W. Deng K. (2019). Development and validation of a nomogram with an autophagy-related gene signature for predicting survival in patients with glioblastoma. Aging (Albany NY) 11 (24), 12246–12269. 10.18632/aging.102566 31844032 PMC6949068

[B45] Xiang Z. Chen X. Lv Q. Peng X. (2021). A novel inflammatory lncRNAs prognostic signature for predicting the prognosis of low-grade glioma patients. Front. Genet. 12, 697819. 10.3389/fgene.2021.697819 34408772 PMC8365518

[B46] Yamaguchi S. I. Ueki A. Sugihara E. Onishi N. Yaguchi T. Kawakami Y. (2015). Synergistic antiproliferative effect of imatinib and adriamycin in platelet-derived growth factor receptor-expressing osteosarcoma cells. Cancer Sci. 106 (7), 875–882. 10.1111/cas.12686 25940371 PMC4520639

[B47] Yan J. Wan P. Choksi S. Liu Z. G. (2022). Necroptosis and tumor progression. Trends Cancer 8 (1), 21–27. 10.1016/j.trecan.2021.09.003 34627742 PMC8702466

[B48] Yoshihara K. Shahmoradgoli M. Martínez E. Vegesna R. Kim H. Torres-Garcia W. (2013). Inferring tumour purity and stromal and immune cell admixture from expression data. Nat. Commun. 4, 2612. 10.1038/ncomms3612 24113773 PMC3826632

[B49] Zhao Z. Liu H. Zhou X. Fang D. Ou X. Ye J. (2021). Necroptosis-related lncRNAs: Predicting prognosis and the distinction between the cold and hot tumors in gastric cancer. J. Oncol. 2021, 6718443. 10.1155/2021/6718443 34790235 PMC8592775

[B50] Zhu N. Hou J. (2020). Assessing immune infiltration and the tumor microenvironment for the diagnosis and prognosis of sarcoma. Cancer Cell Int. 20 (1), 577. 10.1186/s12935-020-01672-3 33292275 PMC7709254

