# Supplementary material for: A novel necroptosis-related lncRNAs signature effectively predicts the prognosis for osteosarcoma and is associated with immunity
Source: Front Pharmacol. 2022 Aug 29;13:944158. doi: 10.3389/fphar.2022.944158 (PMC9465333; doi:10.3389/fphar.2022.944158)
Supplement: Supplementary file 1 [file DataSheet1.DOCX]

**Supplementary Figures**


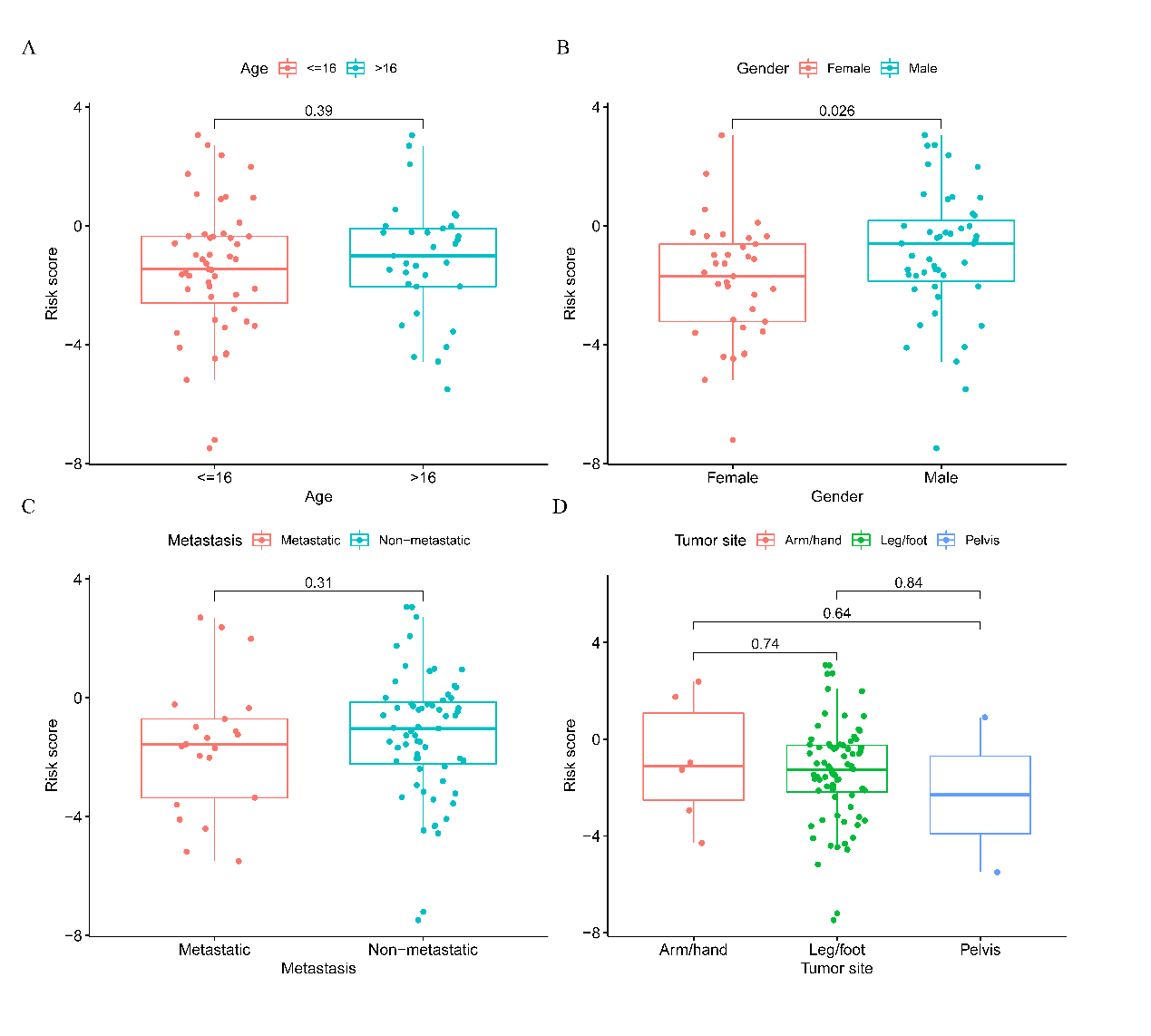


**Figure S1.** The differences in risk scores between different clinical groups.


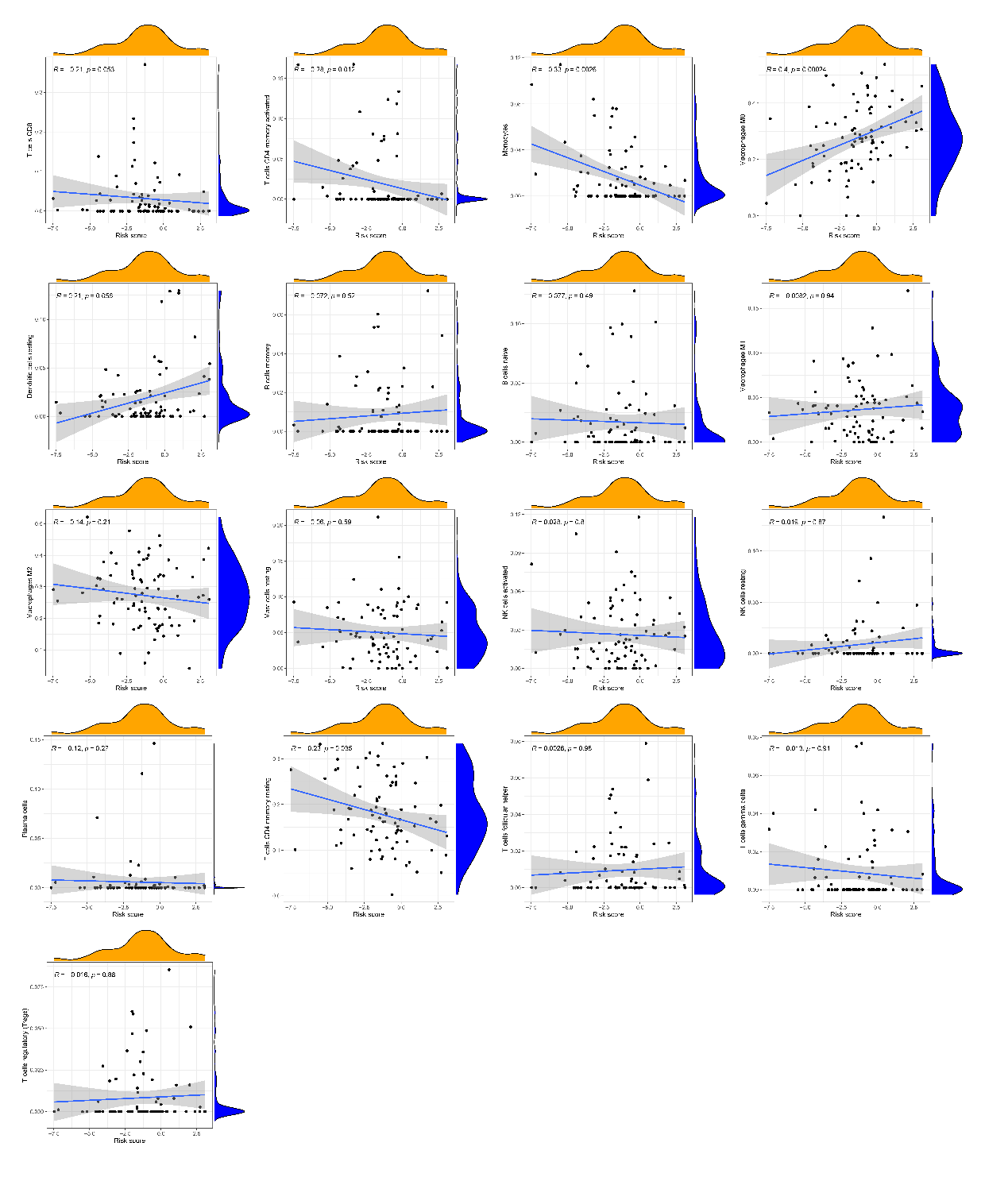


**Figure S2.** The correlation between immune cells and risk score.


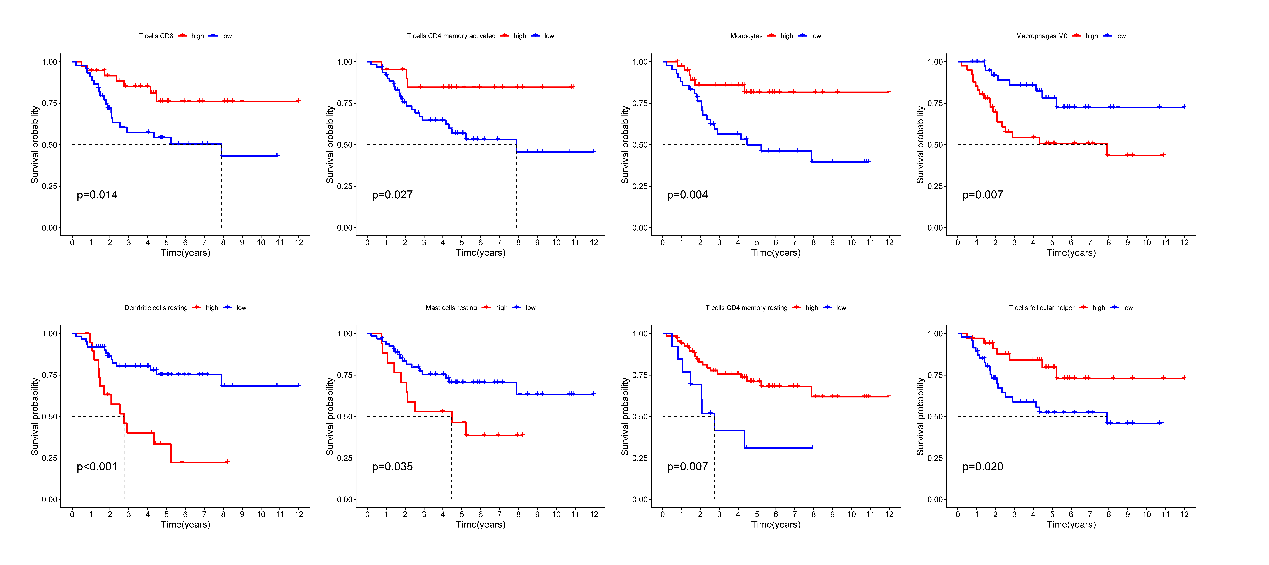


**Figure S3.** K-M survival outcome based on the different proportion of immune cells


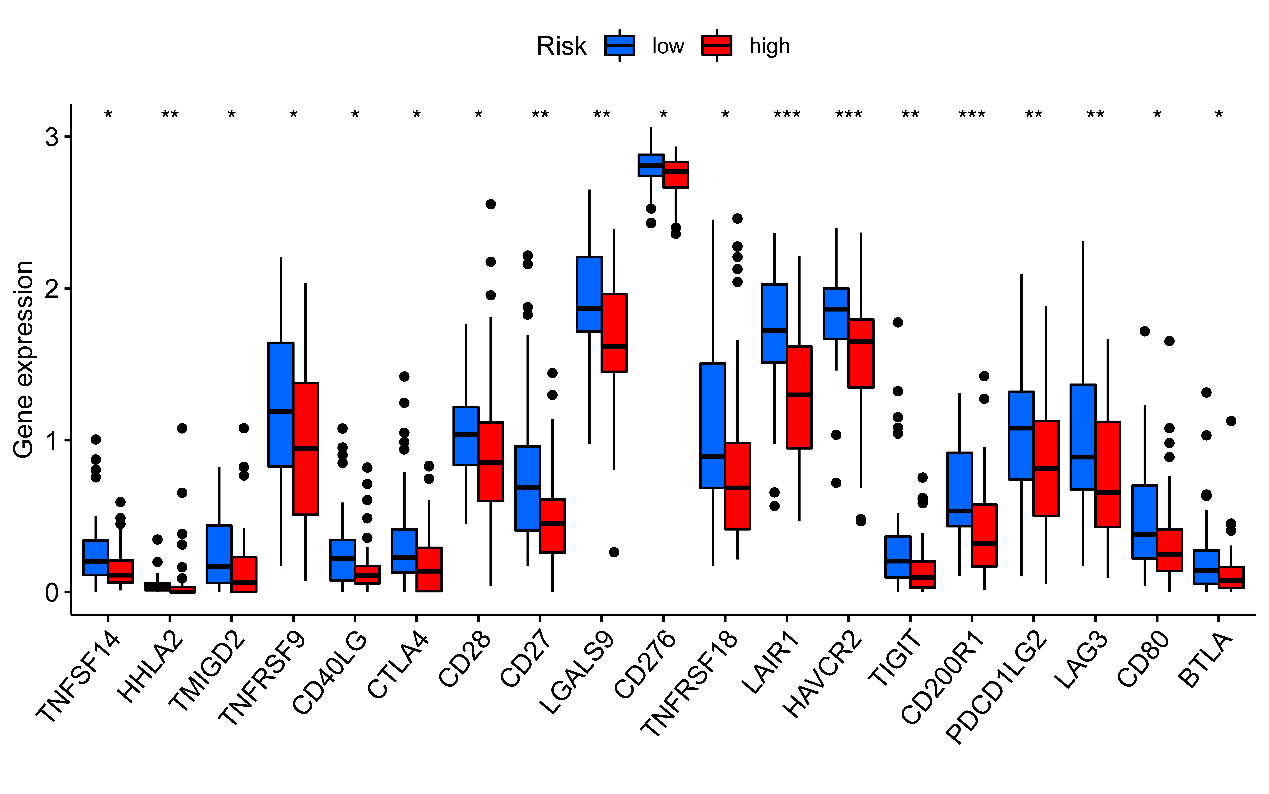


**Figure S4.** Differences in immune checkpoint expression between high- and low-risk groups.
